# Identification of Gait and Personal Factors Associated with Shoe Abrasion Patterns

**DOI:** 10.3390/ijerph191912558

**Published:** 2022-10-01

**Authors:** Jiman Soon, Dae Wook Lee, Gyu Ri Choi, Ki Joon Kim, Sangwoo Bahn

**Affiliations:** 1 Industrial and Management Systems Engineering, College of Engineering, Kyung Hee University, Yongin 17104, Korea; 2 Department of Biomedical Engineering, College of Electronics & Information, Kyung Hee University, Yongin 17104, Korea

**Keywords:** gait, gait pattern, shoe abrasion, walking, body dimension

## Abstract

Shoe abrasion data can be used as major evidence to distinguish suspects, but their actual application in the field is limited due to a lack of associated empirical studies. This study analyzed the significant factors of shoe abrasion by identifying significant differences between gait, personal characteristics, and shoe abrasion patterns. Experiments were conducted on 291 Korean subjects, and data were analyzed using cluster analysis and cross-tabulation analysis with data collected to identify significant factors. As a result, overall, medial abrasion was very rare and would be useful for human identification. The greater the gait characteristics of the knee valgus, the greater the inner abrasion characteristics shown. In the case of knee varus, outer abrasion characteristics occurred more often. Additionally, in the double support phase while walking, the greater the tilt to the left or right, the more the outer parts of the shoes tend to wear out. Men have the characteristic of wearing out the outer side of their shoes more compared to women. Regarding human body dimensions, there were significant differences between the abrasion patterns of the shoes with some body dimensions. The results of this study could be used effectively in the identification of suspects using shoe abrasion patterns.

## 1. Introduction

One of the most crucial factors in the task of assessing the footprints of a crime scene, and whether the suspect’s shoes match, is to identify the abrasion of the shoes. In addition, at the crime scene, the shoe abrasion status is identified using the Footwear impression & Tire imprint Identification System (FTIS) [1] to determine whether the suspect’s foot is flat or leg is limp. As such, crime investigation through shoe abrasion has been applied in many ways. The reason why shoe abrasion is often used in criminal investigations is that each individual has a different environment in which shoes are worn and damaged, so this can be applied to suspect screening [2]. In addition, depending on the different types of walking, such as the degree of tilt of the upper body, the length of the footstep, or the surface of the shoe that touches the ground [3], the abrasion of shoes may vary from person to person. Making data of these different forms of shoe abrasion into the characteristics of different groups, such as individuals, genders, age groups, and body dimensions, could help identify suspects in criminal investigations.

In criminal investigations, the following should be considered in order for shoe abrasion patterns to be useful in terms of personal identification and suspect identification: First, significant results that can be used as evidence must be obtained where the sample size is large enough for generalization [4], so that a large amount of data can be collected to increase the versatility and reliability of the data. Second, given that the evidence at crime scenes remains primarily as video data (CCTV, black box, etc.), among the visually prominent gait parameters in video data, those related to shoe abrasion patterns should be identified. Third, human dimensions, age, gender, and body mass index (BMI), which can identify individuals, should also be considered.

With regard to shoe abrasion, a small number of researchers have conducted research in various fields. Baumfeld et al. [5] surveyed 97 infantrymen, categorizing the abrasion conditions of the boot tread into five types, where they found that the foot valgus/varus and ankle diseases were correlated with shoe abrasion. Finestone et al. [6] examined the heels of the shoes of 76 subjects and found that in general, the shoes had a lot of outer abrasion. Kim et al. [7] also found that each individual had different shoe abrasion patterns by artificially damaging shoe treads for 10 men and women in their 20s, and they identified changes in tread abrasion patterns based on the disappearance of damage patterns. Kang et al. [8] studied a total of 18 subjects to determine how the abrasion form of a shoe affects the walking form and lower extremity joint moment and found that the center of gravity (COG) and Ground Reaction Force (GRF) of the shoe changes depending on the abrasion.

However, the existing research has certain limitations. First, existing shoe abrasion studies investigated between 10 and 100 people, which may not provide sufficient samples to generalize the results. Second, most existing studies used motion tracking sensors, but this differs from image-based analysis in terms of the analysis method and data type. Furthermore, sensor-based analysis may cause some distortion of walking due to the attached sensors and the measurement environment. The utilization of actual walking data is recorded and analyzed through video devices such as CCTV, meaning analysis using video footage can be more effective in actual use. Third, the identification of various personal characteristics, such as age, gender, gait patterns, and body dimensions, which cause shoe abrasion and can be effectively used for the identification of suspects, were not sufficiently considered.

The purpose of this study is to identify the associations between demographic characteristics, gait patterns, body dimensions, and shoe abrasion characteristics and to identify key parameters that can identify individuals by utilizing shoe abrasion. This study conducted experiments on a large number of subjects, up to 291, to compensate for the limitations and increase the utility, and we selected and analyzed 30 visually prominent gait parameters for images taken on the coronal and sagittal axes. The results were analyzed using statistical methods to identify the association between shoe abrasion and more than 30 human characteristics, such as age, gender, body dimensions, BMI, and gait patterns.

## 2. Method

### 2.1. Participants

The experiment was conducted on 291 Korean men and women to identify gait characteristics, human body dimensions, and patterns of shoe abrasion. Among them, 252 people were analyzed, omitting 39 people whose shoe abrasion was not significant enough to be analyzed. The age and gender of the subject were distributed equally to minimize the bias of the results according to the bias of the age and gender of the study. This experiment also involved a much greater number of participants compared with previous studies, demonstrating the validity of the results of the study (see Table 1).

### 2.2. Experimental Environment and Method

**Figure 1:**
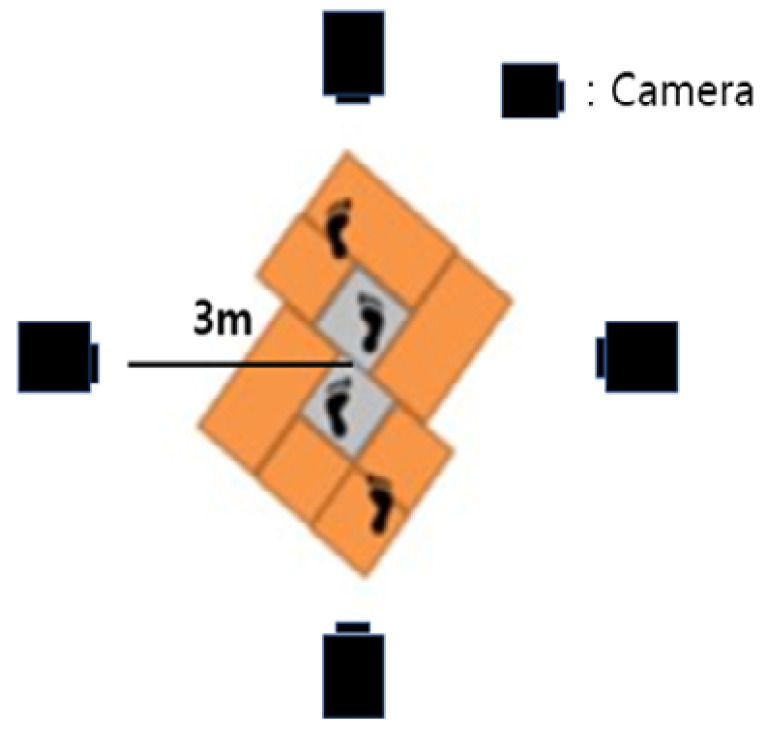
The experimental environment.

Figure 1 shows the walkway with a length of 6 m and a width of 1 m that was installed to observe the gait characteristics of participants. Four video cameras (Panasonic HDC-TM40) were installed outside the walkway in the form of a cross, allowing the gait of participants to be recorded along the sagittal and coronal axes. The participants walked naturally on the constructed walkway, shown in Figure 1. The video was played at the frame rate of 0.01 s to detect the moment of toe-off, heel-strike, and the double support phase. Frontal and sagittal images of the upper body and lower body were then extracted.

### 2.3. Collecting and Classifying Shoe Abrasion Types

Participants brought their worn-out shoes to analyze the abrasion of the shoes according to the human body dimensions and gait characteristics of the participants. After checking the abrasion on the soles, the abrasion of the shoes was classified into five types by referring to the study of Baumfeld et al. [5]. Table 2 shows that the heel is divided into medial, central, and lateral parts to define five types of shoe abrasion.

## 3. Gait Parameters and Human Body Dimensions Parameters

### 3.1. Gait Parameters

**Figure 2:**
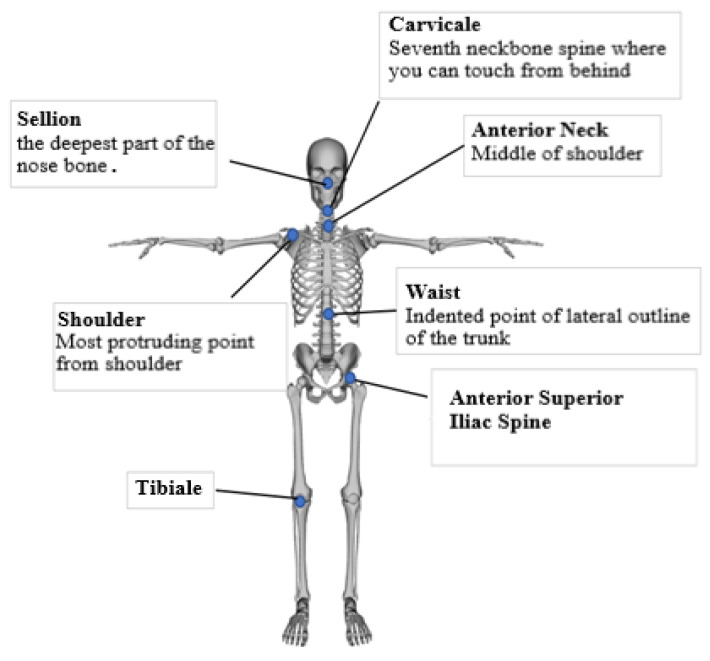
Definition of standard points of the body.

The purpose of this study is to identify the relationship between shoe abrasion characteristics and a person’s personal characteristics, body dimensions, and walking characteristics, and to use them to distinguish individuals by shoe abrasion. To this end, gait parameters that were visually prominent, and which could distinguish individuals, were selected. Each parameter was selected from a comprehensive selection of previous gait studies using visually distinct variables [9,10,11,12,13,14]. Table 3 and Table 4 show the detailed definition and derivation method of each parameter. Additionally, Figure 2 shows the definition of standard points of the human body.

### 3.2. Human Body Dimensions Parameters

Table 5 shows the 11 human body dimensions that were defined to extract the human body dimensions of the participants. These variables are based on the existing human body measurement guidelines suggested by NASA [16], and reports of the measurement of the human body dimensions of Koreans [17]. Each parameter was measured using a Martin Anthropometer.

## 4. Results

### 4.1. Results of the Analysis of Shoe Abrasion Frequency and Gait Pattern

As a result of frequency analysis, both left and right shoes accounted for the majority, with more than 50% of centro-lateral abrasion. Both left and right shoes had the lowest percentage of medial abrasion, with 0.4%, respectively. Centro-medial abrasion came next, with 4.8 and 4.4%, respectively (see Table 6 and Table 7). In other words, overall, as the abrasion part faces inward, the frequency tends to decrease.

### 4.2. Gait Parameters and Shoe Abrasions

Through k-means clustering, 21 gait-related parameters were clustered into three clusters. The left/right tilt-related parameters were also classified into three clusters based on the relatively naturel posture; they were tilted left or right. The statistically significant parameters of the cross-tabulation analysis between each gait parameter and shoe abrasion were the knee varus/valgus and lateral trunk flexion.

The result of the cross-tabulation analysis showed significant differences in the shoe abrasion pattern depending on the knee varus/valgus, without distinction between left and right legs (*p* = 0.00). In the case of the foot varus/valgus, toe-in/toe-out gait, and pelvis abduction/adduction, there were no significant differences in shoe abrasion patterns. The sum of the medial and centro-medial abrasion ratio for the knee valgus was about 18% higher than for the knee varus. Additionally, the sum of the lateral and centro-lateral abrasion ratios was about 21% higher in the knee varus than in the knee valgus (see Table 8). In other words, the knee valgus tends to cause the relative inner abrasion of shoes, while the knee varus tends to cause the outer abrasion of shoes.

The result of the cross-tabulation analysis showed significant differences in the left shoe abrasion pattern, depending on the three groups of lateral trunk flexion at the double support phase (left foot forward) (*p* = 0.00). Participants who showed the left tilt of the upper body when the left foot was forward showed a high lateral abrasion rate (see Table 9).

### 4.3. Gender and Shoe Abrasions

A cross-tabulation analysis was conducted to identify the relationship between gender and shoe abrasion. As a result, the left shoe abrasion pattern (*p* = 0.01) and the right shoe abrasion pattern (*p* = 0.00) differed significantly, depending on gender. Both the left and right shoes of the males had about 15 and 10% higher shoe abrasion rates than those of women, respectively. On the other hand, the medial and centro-medial abrasions of the females’ shoes on both left/right shoes were 10 and 5% higher than those of the males, respectively (see Table 10 and Table 11). In other words, the males had a higher percentage of lateral abrasion of the shoes, while the females tended to have more medial abrasion of the shoe, which corresponded to inner abrasion.

### 4.4. Human Body Dimensions and Shoe Abrasions

The human body dimension parameters were classified into three clusters of small, medium, and large, using k-means clustering analysis. Then, we conducted cross-tabulation analyses to analyze the relationship between the shoe abrasions and a total of 11 human body dimension parameters. The result showed significant differences in the left shoe abrasion patterns (*p* = 0.03), depending on the height of the right ankle (see Table 12). Additionally, the left shoe abrasion patterns (*p* = 0.04) and the right shoe abrasion patterns (*p* = 0.03) differed significantly, depending on the height of the right knee (see Table 13 and Table 14). Overall, the higher the height of the right angle and knee, the more worn the shoes were in the lateral direction.

## 5. Discussion

This study identifies the possibility of distinguishing individuals based on the abrasion patterns of shoes. First, uncommon abrasion patterns based on frequency can be used primarily to identify individuals. Only 0.4% of the 252 subjects were found to have medial abrasion patterns on both left and right shoes. Next, the ratio of centro-medial abrasion was low (left/right: 4.8%/4.4%). Overall, there was a lot of centro-lateral abrasion, and a small percentage of medial abrasion. For medial abrasion and centro-medial abrasion (i.e., inwardly slanted shoe abrasion) among them, in particular, it has been found that abrasion patterns that are inwardly skewed on both feet are very rare. This was similar to the results of a study by Baumfeld et al. [5], who measured 47.1% lateral abrasion and 4.7% medial abrasion in 97 infantrymen. Additionally, the total number of subjects with different forms of abrasion on both feet was 18, accounting for only 6.1% of the total number of participants. The cause of this uneven abrasion could be body imbalance, which also a characteristic of few people. It is also expected to be used for individual identification.

### 5.1. Shoe Abrasion and Gait Patterns

The results of this study showed significant differences in shoe abrasion patterns depending on the knee varus/valgus gait (*p* = 0.00). For those who had knee valgus, the sum of the medial and centro-medial abrasion ratio was about 18% higher than for those who had knee varus. The sum of the lateral and centro-lateral abrasion ratio was about 21% higher in the knee varus than in the knee valgus. This could be because the center of gravity is relatively centered inside the foot at the knee valgus, which shows an X-legged shape. Additionally, the knee varus, which shows an O-legged shape, seems to have a tendency to wear out the outside of the shoe, due to the center of gravity being tilted to the outside of the foot.

There was no significant difference in shoe abrasion patterns depending on the foot varus/valgus. The results of Baumfeld et al. [5], who studied 97 infantrymen, showed significant differences in the shoe abrasion patterns depending on the foot varus/valgus. This seems to be due to the very small percentage of foot valgus in this study. In the study by Baumfeld et al. [5], foot varus accounted for 14%, foot valgus for 40.1%, and neutral for 45.9%. However, when we applied the same foot varus/valgus angle discrimination criteria as Baumfeld et al. [5], the ratio in this study was 90.3, 1.3, and 8.3%, respectively. This could be due to differences in living habits. Most of the population in Eastern countries live sedentary lives, which can cause knee varus [18]. Knee varus legs could cause a foot varus that touches the ground with the outside of the foot.

At the double support phase with the left foot forward while walking, the more the body was tilted in the direction of the front foot, the relatively more types of lateral abrasion there were. The result of the cross-tabulation analysis showed significance at the 0.05 level in the left shoe abrasion patterns depending on lateral trunk flexion (left foot forward) (*p* = 0.00). This can mean that the more the body tilts from side to side at the double support phase of the gait, the more the outer part of the shoe wears out. When stepping in the inside direction of the body when the heel strikes, the degree of tilt of the body can increase. Additionally, when stepping in the inner direction, the outside of the shoe can touch first. Then, the center of gravity is tilted outward, so it is thought that the outside area tends to wear out.

### 5.2. Shoe Abrasion Patterns by Gender

As a result, the difference between men and women in the left and right shoe abrasion patterns was significant (left: 0.01/right: 0.00). Overall, men showed more outer abrasions, while in the case of women, there were more central abrasion patterns than in men. Therefore, it is highly likely that if outer abrasion is present, the subject is male, and that if a shoe is worn inside, the subject is female. This difference in abrasion patterns between genders seems to be due to the following reasons. First, men had an about 5% higher knee varus tendency, which means that men have a tendency to strike the ground with the outside of the foot more than women. Women had an about 6% higher knee valgus tendency than men, which means that women tend to strike the ground with the inside of the foot more than men (see Table 15). These results are consistent with existing results of studying angular differences in knee joints between men and women when walking [19,20]. Second, men have a high rate of toe-out gait (73.8% of all men), while women have a high rate of toe-in gait and neutral gait (68.5% of all women). This result is similar to previous studies that reported that people with varus characteristics in the knee tend to walk in the form of toe-out [21]. In the case of toe-out gait, the outer part of the shoe may be worn more, because the outside of the heel touches the ground first at heel-strike. Additionally, in the case of the toe-in gait and neutral gait, the inner part of the shoe may be worn more, as the middle or inside of the heel touches the ground first at heel-strike.

### 5.3. Shoe Abrasions and Human Body Dimensions

An analysis of the relationship between human body dimensions and shoe abrasion showed a significant relationship between right ankle height/knee height and shoe abrasion. As the right ankle height increases, the ratio of centro-lateral abrasion in the left shoe decreases by about 24%, and the lateral abrasion increases by about 22%. Similar trends have been found in both left/right ankle heights and left/right shoe abrasion. Similar trends were found between the right knee and left and right shoes, but the difference in shoe abrasion patterns was relatively small. These results can be interpreted as a result of men being taller than women on average, resulting in longer lower-extremity-related body dimensions, and men more often tend to have varus in their knees and a toe-out gait [19,20,21]. However, the differences in other parameters, such as height and lower body length, were not significant, so there seems to be a relationship between the ratio of the foot, shin length, and shoe abrasion, but it was difficult to clarify the cause of this in this study. Since there are few related studies, it is difficult to conclude exactly what factors in human body dimensions affect shoe wear. In the future, it seems necessary to reveal more details through additional research on a larger number of people and more balanced objects in terms of human dimensions.

## 6. Limitations of the Study

This study found a difference between left shoe abrasion and right shoe abrasion. This is believed to be due to the asymmetric partial abrasion caused by body imbalance. In this study, we only discussed the overall trend, without considering this. It may be necessary to use left foot and right foot shoes separately to increase their utility as actual evidence. Therefore, it is necessary to find out why left and right feet show different abrasion characteristics and identify related factors through future research. In this study, age and gender were balanced to identify the overall shoe wear characteristics and the main factors that affect them. However, the participation of the elderly was slightly insufficient, and there may be some distortion in terms of the results according to the number of unbalanced elderly participants. In addition, since walking tendency and shoe wear tendency may vary with age, future studies on differences between the young and old may also be beneficial. This study assumed a visual analysis situation, in preparation for future analysis using visual data such as CCTV, and analyzed it based on visually salient variables. Since shoe abrasion is closely related to the precise foot direction and distribution of forces at the time of heel-strike, further analysis will be possible using force plates or motion-tracking sensors that can measure the body movement and dynamics of force more precisely. In addition, due to the lack of participants in the foot valgus, further research on shoe abrasion depending on the foot varus/valgus is needed to conclude the relationship between foot varus/valgus and shoe abrasion.

## 7. Conclusions

In this study, we analyzed the significant correlation between individual human characteristics, gait characteristics, and shoe abrasion types to discriminate individuals. First, after checking the distribution of shoe abrasion shapes through frequency analysis, it was determined that the medial abrasion shape of both feet was very rare and could have a major role in individual identification. Later, cross-tabulation analyses between shoe abrasion types with respect to gait characteristics resulted in different shoe abrasion patterns, depending on the gait characteristics of the knee varus/valgus. In the knee valgus, the inner part of the shoe tends to wear off relatively more, and in the knee varus, the outer part of the shoe tends to wear off. Additionally, at the double support phase while walking, the more tilted the body is to the left or right, the more the outer parts of the shoes tend to wear out. Regarding gender, men show many types of outer abrasion, while women often show wear that is relatively inward compared to men. Regarding the human body dimensions, as the height of the ankle and the height of the right knee increase, the lateral abrasion tends to increase. This study is meaningful in that it correlates with various visually prominent variables that can be analyzed, along with video data such as CCTV data, and is based on the results of a number of subjects compared to existing studies. The results of this study could be utilized when identifying an individual as a reference resource.

## Figures and Tables

**Table 1:** Distribution of participants.

Age		20s	30s	40s	50s	60s	Total
Gender	Male	39	26	24	23	15	127
	Female	37	25	21	27	15	125
Total		76	51	45	50	30	252

**Table 2:** Shoe abrasion types (adapted with permission from Ref. [5]. 2015, Baumfeld et al.).

Abrasion Type	Explanation	Figure
Medial abrasion	The medial part of the shoe is intensively abrased, while the central part is barely abrased.	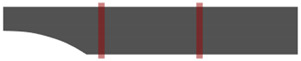
Centro-medial abrasion	The medial and central parts of the shoe are intensively abrased.	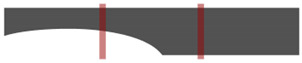
Central abrasion	The central part of the shoe is intensively abrased.	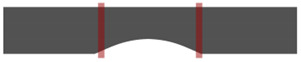
Centro-lateral abrasion	The central and lateral parts of the shoe are intensively abrased.	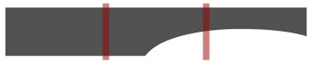
Lateral abrasion	The lateral part of the shoe is intensively abrased, while the central part is barely abrased.	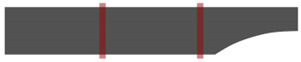

**Table 3:** Definition and derivation method of gait parameters of the lower body (Adapted from Lee et al. 2022 [14], Ergonomic Society of Korea, 2016 [15]).

Gait Parameters	Definition and Derivation Method
Distance between the feet	Definition: The distance between the center of the left and the right foot. Derivation method: Derived by the captured image of the frontal plane at the double support moment. The distance is calculated based on the ratio of the image and the actual distance.
Step length	Definition: Distance between the end point of the front foot and the end point of the back foot. Derivation method: Derived from the captured image of the sagittal plane at toe-off moment. A line is drawn between the end points of the front foot and back foot, and measured, considering the ratio of the image and actual distance.
Number of steps per minute	Definition: Number of steps per minute. Derivation method: The number of steps in the total walk is calculated and divided into the time taken.
Walking speed	Definition: Distance traveled per hour. Derivation method: The ratio of total walking distance to time is calculated.
Swing phase of the left/right foot	Definition: The ratio of feet off the ground to an entire step. Derivation method: The percentage of walking time when the corresponding foot is floating is found.
Stance phase of the left/right foot	Definition: Percentage of feet touching the ground. Derivation method: The percentage of time that the corresponding foot supports the body is found.
Foot angle at heel strike	Definition: The largest angle of the tip of the foot at heel-strike. Derivation method: This is derived from a sagittal image of the heel-strike when the tiptoe is most lifted. A line is drawn between the heel and tiptoe, and the angle between line and ground is measured.
Foot angle at toe-off	Definition: The largest angle of the tip of the foot at toe-off. Derivation method: Derived from a sagittal image of the toe-off when the heel is lifted most. A line is drawn between the heel and tiptoe, and the angle between line and ground is measured.
Knee angle at heel strike	Definition: Knee angle at heel-strike. Derivation method: This is derived from a sagittal image of the heel-strike when the tiptoe is lifted most. Two lines are drawn between the pelvis–knee and knee–ankle, and the angle between the two lines is measured.
Knee angle at toe-off	Definition: Knee angle at toe-off. Derivation method: Derived from a sagittal image of toe-off when the heel is lifted most. Two lines are drawn between the pelvis–knee and knee–ankle, and the angle between the two lines is measured.
Knee Valgus/Varus	Definition: The pattern of knee facing inside (valgus) or outside (varus) the body while walking. Derivation method: This is derived from a frontal image when standing with both feet before starting to walk. Two lines are drawn between the pelvis–knee and knee–ankle, and the exterior angle between the two lines is measured. Considering the measuring error, this measurement is divided into ≥185° as varus, <175° as valgus, and the rest as neutral.
Foot Valgus/Varus	Definition: The pattern of the inside of the foot touching first (valgus), or the outside of the foot touching first (varus), at heel-strike. Derivation method: This is derived from a frontal image when standing with both feet before starting to walk. The results are divided into valgus for those with larger than 1° of valgus, varus for those with larger than 3° of varus, and others as neutral.
Toe-in gait/Toe-out gait	Definition: The direction of the end of toe at heel-strike. Derivation method: This is derived from a frontal image at heel-strike. The results are divided into toe-in gait if the tiptoe faces inside of the body, toe-out gait if the tiptoe faces outside of the body, and the rest as neutral.

**Table 4:** Definition and derivation method of the parameters of the upper body (Adapted from Lee et al. 2022 [14], Ergonomic Society of Korea, 2016 [15]).

Name of Parameter	Definition and Derivation Methods
Pelvis Abduction/Adduction	Definition: The pattern of thigh moves inward (adduction) or outward (abduction). Derivation method: This is derived from a frontal image at the double support phase (mid-point of heel-strike and toe-off). Pelvis adduction is when the front foot crosses or overlaps the end point of the back foot toward the inside of the body in the transverse plane, and pelvis abduction is when the front foot is facing the outside the body, and the knee and toe are placed outside of the pelvis. The rest are neutral.
Lateral flexion of spinal column at the double support phase (left/right foot forward)	Definition: Lateral flexion of the spinal column while the left or right foot stays forward. Derivation method: Derived from a frontal image of the double support phase when the left or right foot stays frontal. Two lines are drawn between the Sellion and Anterior Neck and Anterior Neck and Anterior Waist, and the angle between the two lines is measured. <180° means left tilt of the spinal column, while >180° means right tilt of the spinal column.
Heel strike (toe-off) flexion of the spinal column in the sagittal plane	Definition: Flexion of the spinal column in the sagittal plane at heel-strike (toe-off). Derivation method: This is derived from a sagittal image of the heel-strike (toe-off). A line is drawn between the Lateral Neck and Lateral Waist, and the angle of the line from the vertical is measured.
Lateral trunk flexion at double support phase (left/right foot forward)	Definition: The overall lateral flexion of the trunk in the frontal plane (left or right foot forward). Derivation method: Derived from a captured image of the frontal plane at the double support phase (left or right foot stays frontal). A line is drawn between the Anterior Neck and Anterior Waist, and the angle of the line from the vertical is measured. <180° means left tilted body, while >180° means right tilted body.
Forward/backward trunk bending at heel strike, toe-off	Definition: The overall bending of the trunk in the sagittal plane at heel strike, and toe-off. Derivation method: This is derived by a captured image of the sagittal plane at heel-strike (toe-off). A line is drawn between the Lateral Neck and Pelvis, and the angle of the line from the horizontal is measured.
Shoulder angle in the frontal plane at the double support phase (left/right foot forward)	Definition: Angle due to difference in shoulder height when left or right foot forward. Derivation method: This is derived from a frontal image of the double support phase when the left or right foot stays frontal. A line is drawn between the right and left Lateral Shoulder, and the angle of the line from the horizontal is measured. The result is classified as a higher left shoulder relative to the horizontal right shoulder for less than 180°, and a lower left shoulder for greater than 180°.
Head movements in the frontal plane at the double support phase (left/right foot forward)	Definition: Overall head movements in the frontal plane during the double support phase (left, right foot forward). Derivation method: This is derived from a frontal image of the double support phase when the left or right foot stays frontal. A line is drawn between the Anterior Neck and Sellion, and the angle of the line from the vertical is measured.
Head movements in the sagittal plane at heel strike, toe-off	Definition: Overall head movements in the sagittal plane during the double support phase (left and right foot forward). Derivation method: This is derived from a sagittal image of the heel-strike and toe-off. A line is drawn between the Cervical and Anterior Neck, and the angle of the line from the vertical is measured.

**Table 5:** Human body dimension parameters.

Name of Parameter	Definition
Height, Weight	Height and weight of the body
BMI	Weight in kilograms divided by the square of the height in meters
Ankle height	Height from the floor to the center point of the thinnest part of the ankle.
Foot length	Length from the front of the foot to the tip of the foot
Knee height	Vertical distance from the floor to the Tibiale.
Anterior Superior iliac Spine height	Vertical distance from the floor to the Anterior Superior iliac Spine (ASIS).
Pelvis height	Height from the floor to the pelvic area protruding forward
Pelvis breadth	Width between the left and right ASIS.
Shin height	Height difference between the knee height and ankle height
Femur height	Height difference between the ASIS and knee.

**Table 6:** Frequency analysis results of left shoe abrasion.

Abrasion Pattern	Frequency	Percent (%)	Cumulative Percent (%)
Lateral abrasion	64	25.40	25.40
Centro-lateral abrasion	145	57.54	82.94
Central abrasion	30	11.90	94.84
Centro-medial abrasion	12	4.76	99.60
Medial abrasion	1	0.40	100.00
Total	252	100.00	

**Table 7:** Frequency analysis results of right shoe abrasion.

Abrasion Pattern	Frequency	Percent (%)	Cumulative Percent (%)
Lateral abrasion	73	28.97	28.97
Centro-lateral abrasion	135	53.57	82.54
Central abrasion	32	12.70	95.24
Centro-medial abrasion	11	4.36	99.60
Medial abrasion	1	0.40	100.00
Total	252	100	

**Table 8:** Relationship between the knee varus/valgus and shoe abrasion (X^2^ = 29.23, df = 8, *p* = 0.00).

		Lateral Abrasion	Centro-Lateral Abrasion	Central Abrasion	Centro-Medial Abrasion	Medial Abrasion	Total
Knee varus	Frequency	30	59	7	7	0	103
	%	29.13	57.28	6.80	6.80	0.00	100.00
Neutral	Frequency	95	209	52	12	1	369
	%	25.75	56.64	14.09	3.25	0.27	100.00
Knee valgus	Frequency	6	7	2	4	1	20
	%	30.00	35.00	10.00	20.00	5.00	100.00
Total	Frequency	131	275	61	23	2	492
	percent	26.63	55.90	12.40	4.67	0.40	100.00

**Table 9:** Relationship between the lateral trunk flexion at the double support phase (left foot forward) and left shoe abrasion classification (X^2^ = 40.09, df = 8, *p* = 0.00).

			Lateral Abrasion	Centro-Lateral Abrasion	Central Abrasion	Centro-Medial Abrasion	Medial Abrasion	Total
Lateral trunk flexion at double support phase (left foot forward)	Right-tilted	Frequency	21	91	19	7	0	138
		%	15.22	65.94	13.77	5.07	0.00	100.00
	Neutral	Frequency	5	4	1	0	1	11
		%	45.45	36.36	9.09	0.00	9.09	100.00
	Left-tilted	Frequency	38	50	10	5	0	103
		%	36.89	48.54	9.71	4.85	0.00%	100.00
Total		Frequency	64	145	30	12	12	252
		%	25.40	57.54	11.90	4.76	4.76	100.00

**Table 10:** Relationship between gender and left shoe abrasion classification (X^2^ = 15.04, df = 4, *p* = 0.01).

			Lateral Abrasion	Centro-Lateral Abrasion	Central Abrasion	Centro-Medial Abrasion	Medial Abrasion	Total
Gender	Male	Frequency	42	73	9	3	0	127
		%	33.07	57.48	7.09	2.36	0.00	100.00
	Female	Frequency	22	72	21	9	1	125
		%	17.60	57.60	16.80	7.20	0.80	100.00
Total		Frequency	64	145	30	12	1	252
		%	25.40	57.54	11.90	4.76	0.40	100.00

**Table 11:** Relationship between gender and right shoe abrasion classification (X^2^ = 16.69, df = 4, *p* = 0.00).

			Lateral Abrasion	Centro-Lateral Abrasion	Central Abrasion	Centro-Medial Abrasion	Medial Abrasion	Total
Gender	Male	Frequency	46	70	9	2	0	127
		%	36.22	55.12	7.09	1.57	0.00	100.00
	Female	Frequency	27	65	23	9	1	125
		%	21.60	52.00	18.40	7.20	0.80	100.00
Total		Frequency	73	135	32	11	1	252
		%	28.97	53.57	12.70	4.37	0.40	100.00

**Table 12:** Relationship between the right ankle height and left shoe abrasion classification (X^2^ = 16.66, df = 8, *p* = 0.03).

			Lateral Abrasion	Centro-Lateral Abrasion	Central Abrasion	Centro-Medial Abrasion	Medial Abrasion	Total
Right ankle height	Small	Frequency	16	55	12	3	0	86
		%	18.60	64.00	14.00	3.50	0.00	100.00
	Medium	Frequency	21	63	10	5	0	99
		%	21.20	63.60	10.10	5.10	0.00	100.00
	Large	Frequency	27	27	8	4	1	67
		%	40.30	40.30	11.90	6.00	1.50	100.00
Total		Frequency	64	145	30	12	1	252
		%	25.40	57.50	11.90	4.80	0.40	100.00

**Table 13:** Relationship between the right knee height and left shoe abrasion pattern (X^2^ = 15.72, df = 8, *p* = 0.04).

			Lateral Abrasion	Centro-Lateral Abrasion	Central Abrasion	Centro-Medial Abrasion	Medial Abrasion	Total
Right knee height	Small	Frequency	18	45	11	1	0	75
		%	24.00	60.00	14.67	1.33	0.00	100.00
	Medium	Frequency	24	66	15	10	0	115
		%	20.87	57.39	13.04	8.70	0.00	100.00
	Large	Frequency	22	34	4	1	1	62
		%	35.48	54.84	6.45	1.61	1.61	100.00
Total		Frequency	64	145	30	12	1	252
		%	25.40	57.54	11.90	4.76	0.40	100.00

**Table 14:** Relationship between the right knee height and right shoe abrasion pattern (X^2^ = 16.81, df = 8, *p* = 0.03).

			Lateral Abrasion	Centro-Lateral Abrasion	Central Abrasion	Centro-Medial Abrasion	Medial Abrasion	Total
Right knee height	Small	Frequency	22	37	15	1	0	75
		%	29.33	49.33	20.00	1.33	0.00	100.00
	Medium	Frequency	28	65	13	9	0	115
		%	24.35	56.52	11.30	7.83	0.00	100.00
	Large	Frequency	23	33	4	1	1	62
		%	37.10	53.23	6.45	1.61	1.61	100.00
Total		Frequency	73	135	32	11	1	252
		%	28.97	53.57	12.70	4.37	0.40	100.00

**Table 15:** The result of cross-tabulation analysis between gender and knee varus/valgus (X^2^ = 11.98, df = 2, *p* = 0.00).

Knee Varus/Valgus			Knee Varus	Neutral	Knee Valgus	Total
Gender	Male	Frequency	59	186	3	248
		%	23.79	75.00	1.21	100.00
	Female	Frequency	44	183	17	244
		%	18.03	75.00	6.97	100.00
Total		Frequency	103	369	20	492
		%	20.93	75.00	4.07	100.00

## Data Availability

The data that support the findings of this study are available from the corresponding author, upon reasonable request.
